# High prevalence of diabetes among migrants in the United Arab Emirates using a cross-sectional survey

**DOI:** 10.1038/s41598-018-24312-3

**Published:** 2018-05-01

**Authors:** Nabil Sulaiman, Salah Albadawi, Salah Abusnana, Maisoon Mairghani, Amal Hussein, Fatheya Al Awadi, Abdulrazak Madani, Paul Zimmet, Jonathan Shaw

**Affiliations:** 1 Department of Family and Community Medicine, College of Medicine, University of Sharjah, Sharjah, UAE; 2 Dubai Health Authority, Dubai, UAE; 3 Dubai Hospital, Dubai, UAE; 4 Baker/IDI, Melbourne, Victoria 3004 Australia

## Abstract

In 2011, the United Arab Emirates (UAE) had the 10th highest diabetes prevalence globally, but this was based on data that excluded migrants who comprise 80% of the population. This study assessed diabetes prevalence across the UAE population. A random sample of migrants was recruited from the visa renewal centers. Data were collected using interviews, anthropometric measurements and fasting blood for glucose, lipids and genetic analyses. 2724 adults completed the questionnaires and blood tests. Of these, 81% were males, 65% were ≤40 years old and 3% were above 60 years. Diabetes, based on self-report or fasting plasma glucose ≥7.0 mmol/l, showed a crude prevalence of 15.5%, of whom 64.2% were newly diagnosed. Overall age- and sex-adjusted diabetes prevalence, according to the world mid-year population of 2013, was 19.1%. The highest prevalence was in Asians (16.4%) and non-Emirati Arabs (15.2%) and lowest in Africans and Europeans (11.9%). It increased with age: 6.3% in 18–30 years and 39.7% in 51 to 60 years. Lower education, obesity, positive family history, hypertension, dyslipidemia, snoring, and low HDL levels, all showed significant associations with diabetes. The high diabetes prevalence among migrants in the UAE, 64% of which was undiagnosed, necessitates urgent diabetes prevention and control programs for the entire UAE population.

## Introduction

The Middle East and North Africa (MENA) region has a high prevalence of diabetes mellitus (DM) in adults (10.9%), with particularly high rates reported in the Gulf States. The International Diabetes Federation (IDF) estimated in 2011 that the United Arab Emirates (UAE) had the 10th highest prevalence of diabetes in the world (18.8%), and this was projected to rise to 21.6% by the year 2030^1^. A 1990 study estimated a prevalence of 6% in UAE Emiratis of Bedouin origin (30–64 age group) in which the diagnosis was based on a random capillary blood glucose^2^. A cardiovascular screening program of UAE Emiratis in Abu Dhabi also revealed a high age-standardized prevalence of diabetes (25%) and pre diabetes (30%) defined as fasting blood glucose (FBG) (5.6–6.9 mmol/l) or 2 h post OGTT (7.8–11 mmol/l)^3^.

A survey conducted in both UAE Emiratis and migrants between the years 1999 and 2000 reported a prevalence of 20% (20–64 age group) using the oral glucose tolerance test (OGTT)^2^. A more recent study on 599 migrant women in Al-Ain found that the prevalence of pre diabetes and diabetes was 18.6% and 10.7% respectively, based on HbA1C levels, and that the longer the expats resided in the UAE the more likely they were to develop diabetes^4^. A later survey of adult Emirati citizens living in Al Ain in 2007 reported age-standardized rates among 30–64 year old, of 29.0% for both diagnosed and undiagnosed DM and 24.2% for pre-diabetes^5^. Most countries of the MENA region, in particular countries of the Gulf Cooperative Countries (GCC), have had rapid socioeconomic development and urbanization over the last century following the discovery of oil, leading to epidemiologic and nutritional transition^6^. The economic boom has led to decreased levels of physical activity, increased sedentary behavior and high rates of obesity hence leading to this increasing prevalence of diabetes^4,7^, and its major public health impact on the UAE Emirati and Migrant communities^2^. The UAE has a large and young immigrant workforce, but the burden of diabetes is poorly described in this population group, thus impeding appropriate resource allocation. Although a number of studies have aimed to determine the prevalence of DM in the UAE, the selection of participants has almost always focused on UAE Emiratis. One study focused on both UAE Emiratis and migrants and was conducted more than 15 years ago in 1999 and used the OGTT in reporting T2DM^2^. To our knowledge, there has been no recent study on the prevalence of DM in the Emirati and Migrant population across different emirates of the UAE.

As a result, the UAE National Diabetes and Lifestyle (UAEDIAB) study was conducted to estimate the prevalence of, and risk factors for, type 2 DM. The first phase of this study, concerning the UAE multi-ethnic migrant population, was undertaken during 2013/14. This paper reports results of the first phase of this study.

## Methods

The UAE National Diabetes and Lifestyle Study is a cross-sectional survey designed to investigate the prevalence of diabetes and associated risk factors in UAE Emiratis and Migrants who have been living in the UAE for at least four years. Phase 1 of the study was conducted during 2013 in Dubai, Sharjah and the Northern Emirates. The methods are described in detail elsewhere^8^. Ethical approval for this study was obtained from UAE Ministry of Health on March 14^th^, 2012 and the University of Sharjah ethics committee on 23^rd^ of June, 2010. Every participant read a detailed information sheet and signed an informed consent form to give information as well as blood samples before the interview.

Briefly, the recruitment methods for phase 1 were based on the legal requirement for all UAE migrants to have a medical assessment for visa renewal every 2–3 years at designated Preventive Medicine Departments (PMD). A sample of PMDs was drawn by the UAE National Bureau of Statistics based on size, demographic representation of catchment population, location and numbers of migrants visiting each PMD for visa renewal the year before the survey. A systematic random sample of every 10th PMD attendee was then invited to participate in the study. Assessments included a face-to-face interview to collect demographic data using a general questionnaire. Participants were asked to report their gender, nationality (based on country of origin), date of birth, marital status, residence, highest educational level achieved, family history of diabetes in first degree relatives and lifestyle habits. In addition, they were asked to report their health-related knowledge, attitudes and practices. Following the interview, measurements of weight, height, waist and hip circumference and systolic and diastolic blood pressure were done. In addition, a fasting blood sample was collected after participants’ confirmation that they had been fasting for 8 or more hours and sent to a central laboratory to test for plasma glucose, HbA1c and lipids. Those who didn’t fast were excluded from the study.

Glycemic status was based on self-report, and in separate analyses on FPG and on HbA1c. The HbA1c determination was done in Rashid Diabetes and Research Centre. Lab analysis is based on the turbidimetric inhibition immunoassay (TINIA) for hemolyzed whole blood. Glycohemoglobin (HbA1c) in the sample reacts with anti-HbA1c antibody to form soluble antigen-antibody complexes. Since the specific HbA1c antibody site is present only once on the HbA1c molecule, complex formation does not take place. The polyhaptens react with excess anti-HbA1c antibodies to form an insoluble antibody-polyhapten complex which can be determined turbidimetrically. Hemoglobin(Hb) in the hemolyzed sample is converted to a derivative having a characteristic absorption spectrum which is measured bichromatically during the preincubation phase of the above immunological reaction. HbA1c is calculated from the above two using the formula: HbA1c (%) (DCCT/NGSP) = (HbA1c/Hb) × 91.5 + 2.15. Due to the high specificity this test is not affected by interferences from common hemoglobin variants such as HbS, HbE, HbC, and HbD.

Cut-off values for both tests were defined according to the WHO criteria, whereby FPG < 6.1 mmol/l was normal, 6.1 to 6.9 mmol/l was impaired fasting glucose (IFG), and ≥7.0 mmol/l indicated diabetes. For HbA1c, <6.5% was considered non-diabetic, and ≥6.5% diagnostic of indicated diabetes^9^. Diabetes was further classified into two groups, known diabetes mellitus (KDM) and newly diagnosed diabetes mellitus (NDM). Participants who reported that they had previously been told by a health professional that they had diabetes and were either using glucose-lowering medications or had a FPG ≥7.0 mmol/l or HbA1c ≥6.5% were classified as KDM^10^. NDM was assigned to those participants who reported that they had never been told by a health professional that they had diabetes, but had FPG or HbA1c levels within the diabetes range. Cut-off values were used according to American Diabetes Association to diagnose NDM^11^. The prevalence of total diabetes included all cases (KDM and NDM). BMI was calculated by dividing a participant’s weight in kilograms by the squared height in meters. BMI values below 25 for Arabs and Europeans/Africans and below 23 for Asians were considered normal while BMI values of 30 and above for Arabs and Europeans/Africans, and 27.5 and above for Asians, indicated obesity. Waist circumference was considered normal if it was below 102 cm for males and 88 cm for females for Arabs, below 90 cm for males and 80 cm for females for Asians and below 94 cm for males and 80 cm for European females^12^. Waist to hip ratio below 0.90 for males and 0.85 for females was considered normal for all ethnicities. Snoring and sleep apnea were assessed by asking each subject to report whether they snore loudly and whether anyone had observed them stop breathing during sleep. Hypertension was defined either by a systolic blood pressure of >140 and or a diastolic blood pressure of >90^13^. Triglycerides of >1.7 mmol/L was considered abnormal, while total cholesterol level >5.0 mmol/L was considered high. HDL <1.0 mmol/l for males and <1.3 mmol/l for females was considered low^14^, while LDL was categorized into three groups: <2.59 (optimal), 2.59–3.34 (desirable) and ≥3.34 mmol/l (borderline - high).

All methods described above were performed in accordance with relevant guidelines and regulations and all experimental protocols were approved by the University of Sharjah as well as Ministry of Health Research and Ethics committees.

Analyses were undertaken using Statistical Package for Social Sciences (SPSS) version 22 (IBM Corp, New York). Means with standard deviations (SD), frequency distributions and percentages were used for the univariate descriptive analyses. Between-group comparisons were performed with Chi-square tests for categorical variables. A p-value of 0.05 or less was considered as statistically significant. All variables that were significant in the bivariate analyses were entered into a binary logistic regression model to identify independent correlates of diabetes. Age, gender, employment, ethnicity, education and income were included as covariates in the regression model. The Hosmer and Lemeshow test was used to check for the goodness of fit of data and the Omnibus test was used to assess whether the logistic regression model significantly explained the variance in the outcome variable. The direct standardization method was used to calculate the prevalence of diabetes adjusted by age and sex to the world mid-year population of 2013^15^.

Data will be available on request from the principle investigator.

## Results

**Figure 1:**
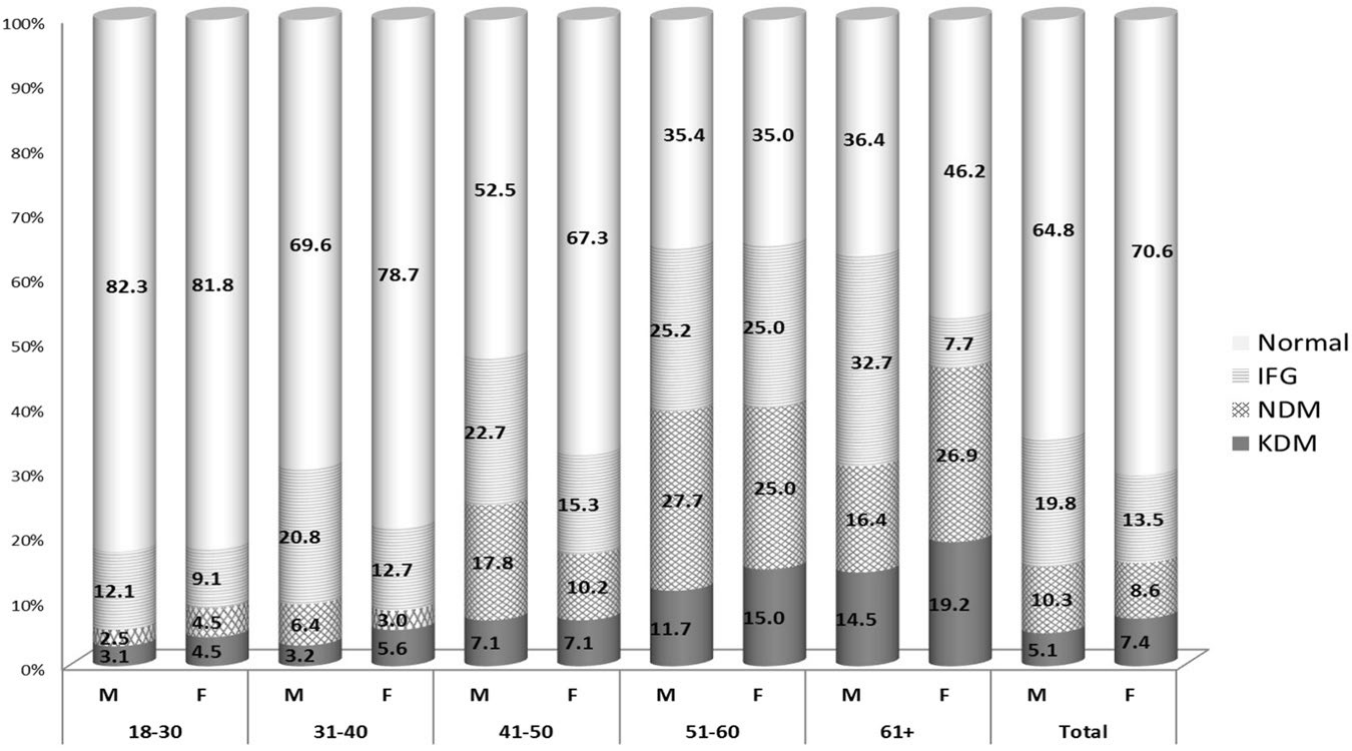
Distribution of Diabetes Status by age and Gender.

The total number of migrants recruited was 2724 (response rate = 68%). The response rate was slightly lower in the Sharjah Emirate due to language barriers which were later resolved by including data collectors who were fluent in Hindi. Women comprised 19% of the study sample. Migrants were mainly South Asian (mostly Indians and Pakistanis), and non-Emirati Arabs (mostly Egyptians, Syrians, Jordanians, Iraqis, Palestinians and Sudanese), with the majority being males and aged under 40 years (Table 1). The mean age of participants was 38 (SD = 10.34) ranging between 18 and 80 years. The crude prevalence rates of total diabetes (including KDM and NDM) and of IFG were 15.5% and 18.6% respectively. The adjusted prevalence (95% CI) of total diabetes, after standardizing to the world mid-year population of 2013, was 19.1% (17.6–20.5%) and of IFG was 15.3% (14.0–16.7%). Overall, the percentage of diabetes that was undiagnosed was 64.2%. The prevalence of diabetes increased with age, from 6.3% in the 18–30 year olds to 39.7% in those aged 51–60 (Table 2). The prevalence of total diabetes was similar in both genders, though KDM was slightly higher in females and NDM was higher in males (Fig. 1). IFG prevalence was significantly higher among males than females (19.8% vs 13.5%, p < 0.004). Overall, there were no differences between ethnic groups, but when stratified by age, there was a statistically significant difference in the 18–30 age group, between non-Arab Asians (7.2%) and Arabs (3.1%) (p = 0.021).

**Table 1:** Demographic Characteristics of Migrant Study Population.

Sex	N	%
Male	2204	81.1
Female	515	18.9
**Age**		
18–30	689	25.3
31–40	1080	39.6
41–50	607	22.3
51–60	267	9.8
≥61	81	3.0
**Emirate**		
Dubai	1192	43.8
Fujeirah	155	5.7
Ajman	351	12.9
Ras Al Khaimah	308	11.3
Sharjah	626	23.0
Um Al Quwain	92	3.4
**Ethnicity**		
Asians non-Arabs	1727	70.7
Arabs	633	25.9
Europeans	56	2.3
Africans	28	1.1
**Marital status**		
Married	2184	80.2
Single	504	18.5
Separated/Divorced/Widowed	34	1.2
**Highest Level of Education**		
Never attended school	92	3.4
Primary school	294	10.8
Secondary school	1019	37.4
Diploma	342	12.6
University Bachelor degree	756	27.8
Post-graduate diploma	108	4
Masters / Doctorate degree	112	4.1
**Occupation**		
Managers & professional	646	23.7
Technical workers	665	24.4
Skilled workers	142	5.2
Elementary occupations	511	18.8
Unemployed	126	4.6
Not specified	634	23.3
**Smoking status**		
Current smoker	551	20.2
Ex-smoker	92	3.4
Non-smoker	2080	76.4
**BMI** ^*^		
Normal	575	21.9
Overweight	1172	44.6
Obese	881	33.5
Waist circumference**		
None-obese	997	41.1
Obese	1428	58.9
Waist to Hip ratio***		
None-obese	883	36.5
Obese	1538	63.5
**Hypertension**		
No	1848	68.1
Yes	866	31.9
**Total**	**2724**	**100**

^*^BMI was categorized according to the following criteria (Normal: Arabs & Europeans – BMI < 25 Asians–BMI < 23; Overweight:Arabs & Europeans – 25 ≤ BMI < 30, Asians – 23 ≤ BMI < 27.5; Obese: Arabs & Europeans – BMI ≥30, Asians – BMI ≥27.5).

**Waist Circumference was categorized according to the following criteria (obese: Arabs – males WC ≥102 cm & females ≥88; Asians – males WC ≥90 cm & females ≥80; Europeans – males WC ≥94 cm & females ≥80).

***Waist to Hip ratio (WHR) was categorized according to the following criteria (WHR for males ≥0.90 cm & females ≥85 indicated Obesity for all ethnicities).

**Table 2:** Prevalence and Risk of Diabetes by Demographic Factors.

Variable	N	Diabetes Prevalence (%)		Odds ratio of Diabetes (95% CI)			
				Age-sex adjusted		Adjusted for multiple factors*^	
**Age**							
18–30	686	6.3	*p* < *0.0005*	Reference group		Reference group	
31–40	1077	9.4		1.55 (1.07–2.24)	P = 0.021	1.37 (0.87–2.15)	P = 0.167
41–50	606	23.6		4.62 (3.22–6.63)	P < 0.0005	4.89 (3.17–7.53)	P < 0.0005
51–60	267	39.7		9.74 (6.57–14.46)	P < 0.0005	11.09 (6.87–17.91)	P < 0.0005
61+	81	35.8		8.35 (4.81–14.47)	P < 0.0005	7.20 (3.53–14.66)	P < 0.0005
**Gender**							
Male	2199	15.4	*P* = *0.749*	Reference group		Reference group	
Female	513	16.0		0.98 (0.74–1.30)	P = 0.893	0.76 (0.50–1.14)	P = 0.187
**Employment**							
Managers & Professionals	643	14.5	*P* = *0.013*	Reference group		Reference group	
Skilled workers	805	14.0		1.17 (0.86–1.61)	P = 0.316	1.09 (0.73–1.63)	P = 0.684
Elementary & unemployed	636	19.3		1.76 (1.29–2.42)	P < 0.0005	1.53 (0.98–2.40)	P = 0.061
**Ethnicity**							
Arabs non-nationals	631	15.2	*P* = *0.471*	Reference group		Reference group	
Asians non-Arabs	1723	16.4		1.22 (0.93–1.59)	P = 0.158	1.20 (0.87–1.65)	P = 0.267
Europeans & Africans	84	11.9		0.71 (1.29–2.42)	P = 0.360	0.53 (0.21–1.32)	P = 0.174
**Education**							
Below university	1742	16.1	0.254	1.13 (0.90–1.43)	P = 0.287	0.95 (0.68–1.32)	P = 0.740
University & above	974	14.5		Reference group		Reference group	
**Income (AED per year)**							
Less than 96000	1815	16.0	*0.721*	1.31 (1.02–1.68)	P = 0.033	1.04 (0.71–1.50)	P = 0.857
> = 96000	750	15.5		Reference group		Reference group	

^*^Adjustment was made for age, gender, employment, ethnicity, education and income.

^Omnibus test model (χ^2^ = 187.512, p < 0.0005); Cox & Snell R^2^ = 0.100 & Nagelkerke R^2^ = 0.169; Hosmer & Lemeshow Test (χ^2^ = 1.250, df = 8, p = 0.996).

Regression analysis showed that age, obesity (but not overweight), family history of diabetes, snoring and LDL level ≥3.34 mmol/L were independently associated with diabetes (Table 3).

**Table 3:** Prevalence and Adjusted Odds ratio of Diabetes by Biomedical Risk Factors.

Variable	N	Diabetes Prevalence (%)		Odds ratio of Diabetes (95% CI)			
				Age-sex adjusted		Adjusted for multiple factors*^	
**Smoking**							
Yes	546	15.4	*p* = *0.912*	1.01 (0.77–1.33)	p = 0.927	0.94 (0.66–1.34)	p = 0.732
No	2170	15.6		Reference group		Reference group	
**Obesity**							
Normal	574	10.1	p < 0.0005	Reference group		Reference group	
Overweight	1169	12.6		1.10 (0.79–1.54)	p = 0.569	1.010 (0.64–1.57)	p = 0.998
Obese	879	23.5		1.94 (1.39–2.69)	p < 0.0005	1.65 (1.05–2.59)	p = 0.030
**Family History of DM**							
Yes	687	24.0	p < 0.0005	2.23 (1.77–2.82)	p < 0.0005	2.37 (1.74–3.23)	p < 0.0005
No	2029	12.7		Reference group		Reference group	
**Vigorous/Moderate Intensity Activity at Work**							
Yes	457	16.2	p = *0.672*	1.03 (0.77–1.38)	p = 0.842	0.94 (0.65–1.35)	p = 0.727
No	2259	15.4		Reference group		Reference group	
**Watching TV for less than one hour per day**							
Yes	1077	17.5	p = 0.015	1.25 (0.99–1.56)	p = 0.054	1.11 (0.83–1.49)	p = 0.471
No	1585	14.1		Reference group		Reference group	
Snoring							
Yes	509	25.0	p < 0.0005	1.61 (1.25–2.08)	p < 0.0005	1.44 (1.04–1.98)	p = 0.028
No	2009	13.2		Reference group		Reference group	
**HDL**							
< 1 for males & < 1.3 for females	1001	18.7	p = 0.001	1.56 (1.25–1.95)	p < 0.0005	0.76 (0.56–1.05)	p = 0.093
≥1 for males & ≥1.3 for females	1711	13.7		Reference group		Reference group	
**LDL**							
<2.59	490	15.7	p = *0.924*	Reference group		Reference group	
2.59–3.34	967	15.8		0.94 (0.68–1.29)	p = 0.685	0.88 (0.57–1.35)	P = 0.551
≥3.34	1260	15.2		0.82 (0.61–1.12)	p = 0.211	0.53 (0.29–0.94)	P = 0.031
**Triglycerides**							
<1.7	1718	12.4	p < 0.0005	Reference group		Reference group	
≥1.7	998	20.8		1.74 (1.39–2.17)	p < 0.0005	1.34 (0.98–1.84)	P = 0.065
**Total Cholesterol**							
<5.0	1312	14.7	p = *0.253*	Reference group		Reference group	
≥5.0	1405	16.3		1.01 (0.81–1.26)	p = 0.908	1.36 (0.85–2.17)	P = 0.196
**Hypertension**							
Yes	864	21.1	p < 0.0005	0.80 (0.63–1.01)	p = 0.055	0.99 (0.73–1.35)	p = 0.977
No	1843	13.0		Reference group		Reference group	
≥1 for males & ≥ 1.3 for females	1711	13.7		Reference group		Reference group	

*Adjustment was made for age, gender, employment, ethnicity, education and income.

^Omnibus test model (χ^2^ = 247.577, p < 0.0005); Cox & Snell R^2^ = 0.138 & Nagelkerke R^2^ = 0.232; Hosmer & Lemeshow Test (χ^2^ = 4.544, df = 8, p = 0.805).

**Figure 2:**
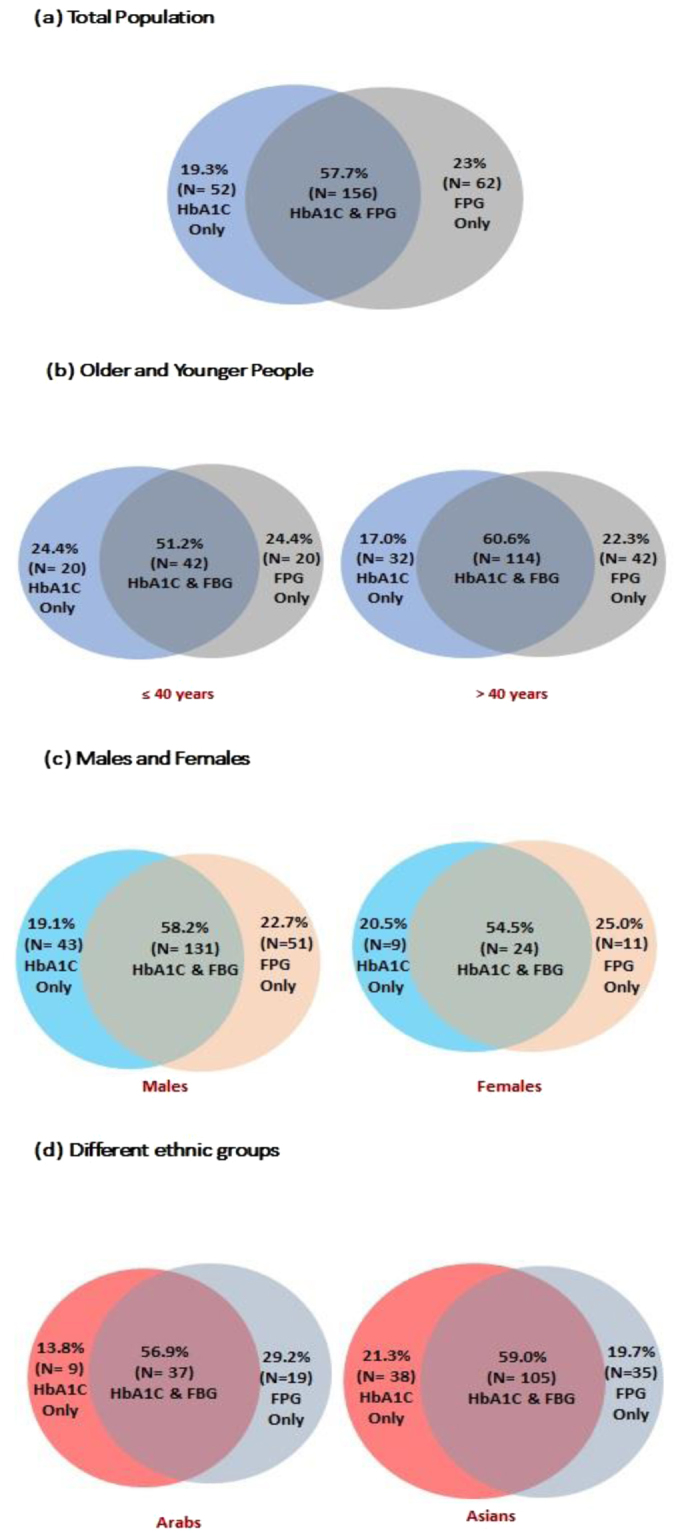
Distribution of cases of newly diagnosed diabetes according to FpG FPG and HbA1c in (**a**) Total total population, (**b**) older and younger people, (**c**) males and females and (**d**) Arabs and Asians.

Figure 2 shows the concordance/discordance between FPG and HbA1c results among those with NDM. The numbers of people identified as having NDM were similar whether FPG or HbA1c were used as the diagnostic criterion. Furthermore, most people with diabetes by either parameter were diabetic on both parameters. Examining sub-groups, the prevalence of NDM was similar by FPG and HbA1c for older and for younger participants, for males and for females, and for Arabs, Asians and Europeans.

## Discussion

Phase I of the present UAEDIAB study provides the most recent estimate of the prevalence of type 2 diabetes among the rapidly changing UAE Migrants, who comprise 80% of the total UAE population^16^. The age distribution of the study sample is comparable to that of the UAE migrant population reported in the last UAE census conducted in 2005. Females were under-represented in this study, compared to the census migrant population (18.9% vs 27.2%) in 2005^16^. Our study found a high prevalence of diabetes in UAE Migrants (19.1%) who are mainly Asians and non-UAE Arabs. Most of the persons with diabetes (64.2%) were undiagnosed prior to the study. This has direct implications for national policy development to screen the adult population.

Our study revealed a high diabetes prevalence in Asians (16.4%) of whom the majority are from India and Pakistan. This is higher than the prevalence in their countries of origin^17–19^ but lower than the 19.1% reported by the 1999–2000 UAE National survey which used the OGTT^2^. Both South Asian immigrants and individuals living in their home countries have a rapidly increasing diabetes prevalence^18^, suggesting that lifestyle and environmental factors may be key factors^14^. However, while aging, urbanization, and associated lifestyle changes are being suggested as key determinants for these rapid increases, an adverse intrauterine environment and the resulting epigenetic changes could also contribute in many developing countries in Asia^20,21^.

The prevalence in Non-Emirati Arabs, who were mainly from Egypt, Syria, Iraq, Jordan, Sudan, Palestine and Lebanon, was 15.2%. This is comparable to the 1999–2000 UAE National Survey which reported a prevalence range of 12.7% to 19.1%^2^.

Among those aged 18–30 years, the prevalence of T2DM was 6.3% and of IFG was 11.7%; evidence indicates that there is an emerging epidemic of T2DM in the youth^16^. It is notable that age had a stronger relationship with the prevalence of diabetes than with the prevalence of IFG.

Obesity is one of the main consistent explanations to the high rate of diabetes in the UAE and other Gulf countries^22^ and several studies have shown a strong association between obesity as a risk factor and the development of diabetes^23,24^. Interestingly, there was no increase in the prevalence of diabetes among those who were overweight. This is consistent with a previous UAE study^2^, and has important public health and clinical implications. It suggests that the focus needs to be on obesity, rather than overweight, and that moving from obesity to overweight can deliver the full benefit of weight loss.

There are other risk factors that were also associated with T2DM in this study population. The highest prevalence rates were seen in participants who were unemployed or had elementary (unskilled) occupations. Those who had either not attended school or only attended primary school also had a greater risk of having diabetes. A number of studies have shown a higher risk of T2DM in individuals with lower education attainment and income^25^ and there is evidence that in high-income countries specifically, low socioeconomic status is related to T2DM^26^. Individuals with lower income levels may have low health status indicators^27^, limited resources, lesser range of affordable food choices, and more stress^28^. Additionally they may not be able to afford health related activities^29,30^.

Other independent risk factors for diabetes included age, gender, family history of diabetes, and dyslipidemia. This is consistent with an earlier study conducted in the UAE population^31^.

One major strength of this study is that it provides recent data on a national level about diabetes prevalence and its risk factors in UAE based on FPG. The main limitation of this study was the difficulty in convincing participants to fast for more than 8 hours, which proved to be challenging. Similarly trying to accommodate about 30–45 minutes extra time for body measurements and interview, for individuals coming at the last minute to renew their visa proved to be difficult at times. These factors contributed to a modest response rate of 68%, which although quite good for health surveys, may limit the certainty with which findings can be generalised.

## Conclusions

This study showed that the very high diabetes prevalence previously reported in the Emirati population in the UAE is also observed in the much larger migrant populations. Had an OGTT been performed, the prevalence would have been even higher^32^. Almost two thirds of people with diabetes were undiagnosed. By bringing to the fore the burden of diabetes, this study has showcased the urgency by which this health problem needs to be addressed and demonstrates the urgent need for better preventative and screening measures nationally. Appropriate screening strategies need to be established and preferably carried out in the primary health care settings. Importantly, there needs to be a focus on meeting the health needs of migrants in the UAE. This will ultimately lead to prevention of complications and long term consequences, which already are causing a huge burden on the UAE health care system.
